# Sea Buckthorn Proanthocyanidins are the Protective Agent of Mitochondrial Function in Macrophages Under Oxidative Stress

**DOI:** 10.3389/fphar.2022.914146

**Published:** 2022-07-08

**Authors:** Keshan Liu, Wenxia Li, Michael Yuen, Tina Yuen, Hywel Yuen, Min Wang, Qiang Peng

**Affiliations:** ^1^ College of Food Science and Engineering, Northwest A&F University, Yangling, China; ^2^ Puredia Limited, Xining, China

**Keywords:** sea buckthorn, proanthocyanidins, antioxidant, mitochondrial function, RAW264.7 cells

## Abstract

Sea buckthorn proanthocyanidins (SBP) are the most important antioxidant components of sea buckthorn, which are widely used in functional foods and cosmetics. Studies have shown that SBP have significant protective effects on macrophages against oxidative stress induced by hydrogen peroxide (H_2_O_2_). However, the mechanism remains uncertain. In the present study, we explored the effects of SBP on mitochondrial function and the mechanism of their protective effects against oxidative stress in cells. Our results showed that SBP could increase mitochondrial membrane potential, inhibit mPTP opening, reduce mitochondrial swelling, and enhance mitochondrial synthesis and metabolism. Thus, they alleviated oxidative damage and protected the cells against mitochondrial function. Western blot analysis showed that SBP had a protective effect on RAW264.7 cells by activating the AMPK-PGC1α-Nrf2 pathway. These results showed that SBP alleviated mitochondrial damage and dysfunction caused by oxidative stress. This study revealed the mechanism of SBP in reducing oxidative damage and provided a theoretical basis for further research on natural bioactive compounds to exert antioxidant activity and prevent arteriosclerosis and other diseases.

## Introduction

Sea buckthorn (Hippophae rhamnoides L.) belongs to the Solanaceae family and is widely grown in Asia, Europe, and North America. Sea buckthorn is a plant with strong vitality. It can resist drought, wind, and sand and grow in saline-alkali land. In China, Sea buckthorn is categorized as a “Medicine and Food Homology”. It is a famous traditional Chinese herb used to treat indigestion, bronchitis, cardiovascular disease, irregular menstruation, and menopause. Sea buckthorn contains many nutritional and bioactive compounds such as proanthocyanidins, flavonoids, polyphenols, polyunsaturated fatty acids, carotenoids, sugar alcohols, and superoxide dismutase and phytosterols, which have cardiovascular, anti-inflammatory, immunological regulation and antioxidants effects (Wang et al., 2021). Sea buckthorn contains remarkably high amounts of antioxidants. Thus, it has an important nutritional value (Ciesarová et al., 2020). One of the most important antioxidants in sea buckthorn, proanthocyanidins, contributes to the nutritional benefits of sea buckthorn products.

Proanthocyanidins are polyphenols in plant foods that have many health benefits, including cancer prevention, cardiovascular protection, and diabetes prevention. The composition and degree of polymerization of proanthocyanidins determine their digestion, absorption, and biological activity. The absorption properties of oligomeric proanthocyanidins monomers and dimers are better in the small intestine than polymers (DP > 4) (Zhang et al., 2016). Our previous studies isolated sea buckthorn oligomeric proanthocyanidins (SBP) from the fruits of sea buckthorn produced in China’s Qinghai Province. Chemical composition analysis showed that SBP was composed of epicatechin gallate, b-type proanthocyanidins, gallocatechin-catechin, and gallocatechin dimer (Zhu et al., 2021). Oxidative stress is closely related to cardiovascular problems (Shields et al., 2021). Mitochondria are intracellular energy factories associated with redox signal transduction, apoptosis regulation, and gene expression regulation. They are involved in developmental biology, genetics, aging, cardiovascular diseases, and other aspects of the organism.

Mitochondrial damage is one of the main factors causing atherosclerosis. Impaired mitochondrial respiratory function results in inhibition of electron transfer, low-density lipoprotein oxidation, loss of scavenging agents, and permanent dysfunction of cardiomyocytes (Chakrabarty et al., 2018). SBP also protect macrophages against oxidative stress induced by hydrogen peroxide (Zhu et al., 2021). However, the mechanisms by which SBP protect macrophages under oxidative stress conditions remain uncertain.

The present study investigated whether SBP treatment could alleviate mitochondria dysfunction induced by oxidative stress. In addition, the effect of SBP on mitochondrial morphology was investigated using transmission electron microscopy (TEM). Furthermore, the underlying mechanisms by which SBP alleviated H_2_O_2_-induced mitochondrial dysfunction in RAW 264.7 cells were studied. This study illuminated the role of SBP on mitochondria recovery after oxidative stress and provided a mechanistic link between SBP treatment, mitochondrial health, and antioxidative function.

## Materials and Methods

### Materials and Reagents

Sea Buckthorn Proanthocyandins (SBP), were provided by Puredia Limited (Qinghai, China). SBP were extracted from the fruits of sea buckthorn by water extraction and macroporous resin column chromatography, trademarked as CyanthOx™. The purity of SBP was 91.5% comparing with the standard of proanthocyanidins. They were mainly composed of (-)-epicatechin gallate, b-type proanthocyanidins, (+)-gallocatechin-(+)-catechin, and (+)-gallocatechin dimer (Chakrabarty et al., 2018). Fetal bovine serum was purchased from PAN-Biotech GmbH (Aidenbach, Germany). RPMI1640 medium was purchased from BasalMedia Technologies (Shanghai, China). Mito-Tracker Green (C1048) and MitoTracker Red CMXRos (C1049B) were obtained from the Beyotime Institute of Bio-technology (Shanghai, China). Mitochondrial Permeability Transition Pore Assay Kit (C2009S), Cell Mitochondria Isolation Kit (C3601), and ATP Assay Kit (S0026) were purchased from Beyotime Biotechnology, Shanghai, China. The glucose quantification kit was purchased from Applygen, Beijing, China. The antibodies of HO-1, p-AMPK, and p-Nrf2 were purchased from Cell Signaling Technology (Shanghai, China). PGC-1α was purchased from Bioss Inc. (Woburn, America).

### Cell Culture

RAW 264.7 cells, a macrophage cell line, were purchased from the American Type Culture Collection (ATCC, Manassas, VA, United States). The macrophages were cultured in RPMI1640 medium with 10% FBS, 100 U/mL penicillin, and 100 μg/ml streptomycin in a 5% CO_2_ atmosphere at 37°C. Cells were passaged when they became 70–80% confluent.

### Cell Treatment

The RAW 264.7 cells (1.0 cells/ml × 10^5^ cells/ml) were incubated with different concentrations of SBP (0, 25, 50, and 100 μg/ml) for 4 h, followed by the addition of 800 μmol/L H_2_O_2_ and treated for another 4 h.

### Measurement of Mitochondrial Membrane Potential

RAW264.7 cells (2.0 cells/ml × 10^5^ cells/mL) were incubated in 6-well tissue culture plates at 37°C for 24 h, then removed from the medium and exposed to different concentrations (25, 50, 100 μg/ml) of SBP solution for 4 h. Subsequently, 800 μmol/L of H_2_O_2_ was added to sample groups and incubated for another 4 h. The parameters of mitochondrial membrane potential (MMP) and Malondialdehyde (MDA) were determined using the corresponding kits (Beyotime Institute of Biotechnology, Shanghai, China). The test cells were added with 100 µL of 5 μg/ml JC-1 for 30 min and then quantified by the fluorescence microscope and microplate reader.

### Observation of Mitochondria Morphology

The distribution of mitochondria was analyzed using Mito-Tracker Red CMXRos (Mitochondrial red fluorescent probe). Cells were incubated in the medium containing 100 nm Mito-Tracker Red CMXRos for mitochondrial staining at 37°C for 40 min. After washes with cell culture medium, cells were observed by fluorescence microscopy (IX71-F22FL/PH, Olympus, Tokyo, Japan) (Maeda et al., 2004). Transmission electron microscopy was used to observe the microstructure of mitochondria. Cells were fixed in 2.5% glutaraldehyde for 2 h, then washed by PBS (phosphate-buffered saline) and fixed again in 1% osmium tetroxide for 1 h. Gradient dehydration was performed before immersing into epoxy resin overnight at 37°C and polymerizing at 60°C for 48 h. After that, the samples were sliced and stained with 2% uranium dioxide acetate dye and lead citrate, respectively. Finally, the samples were washed and dried before observation by Transmission electron microscopy (JEM-1230, JEOL, Tokyo, Japan).

### Measurement of Mitochondrial Permeability Transition Pore and Swelling

Mitochondrial permeability was measured by monitoring the fluorescence of mitochondrial entrapped calcein (MPTP Assay Kit). Calcein AM staining solution and fluorescence quenching working fluid were added at 37°C followed by incubation in the dark for 30 min after PBS washing twice. Then, a fresh culture solution was added to incubate the cells for another 30 min in the dark at 37°C. Finally, the cells were washed with PBS twice before observing by fluorescence microscopy (IX71-F22FL/PH, Olympus, Tokyo, Japan) (Petronilli et al., 1999). Mitochondrial swelling was measured by a microplate reader at 540 nm. The mitochondria were isolated by a mitochondria isolation kit according to the manufacturer’s instructions. The mitochondrial protein concentration was measured by bicinchoninic acid assay and 10 μL CaCl2 (20 mm) was mixed with cellular mitochondria (500 nmol mg^−1^ mitochondrial protein) to induce mitochondrial swelling (Luth et al., 2014; Han et al., 2021).

### Determination of Mitochondrial Mass by Flow Cytometry Analysis

Mito-Tracker green was applied to characterize the mitochondrial morphology in RAW 264.7 cells. Briefly, Mito-Tracker green stock solution (1 mm) was prepared with anhydrous dimethylsulfoxide and diluted with Hank’s Balanced Salt Solution. The cells were incubated with Mito-Tracker green staining solution (200 nm) at 37°C for 20 min. The staining solution was then removed, and fresh culture solution was added to the wells. The cells were determined by a flow cytometer (CyFlow Cube 6, Partec, Munich, Germany) at 488 nm of excitation wavelength and 516 nm of emission wavelength. Flowjo software was applied to the quantification of the number and individual volume of the mitochondria.

### Measurement of Mitochondrial Adenosine Triphosphate Synthesis and Glucose Utilization

ATP levels were measured using the ATP Assay Kit. Briefly, the collected cells were lysed and centrifuged at 12,000 g for 5 min at 4°C. In 12-well plates, 100 μL of each supernatant was mixed with 100 μL ATP working dilution. Luminescence was detected using a microplate reader (PowerWave XS, Bio-Tek, Vermont, US). The ATP level was presented as nanomoles per milligram of protein. The glucose utilization of cells was determined by the glucose quantification kit. The glucose utilized over the 24 h period was calculated by subtracting the glucose concentration at 24 h from that at 0 h (Chen et al., 2018).

### Western Blot Analysis

Briefly, RAW264.7 cells were washed with PBS (ice-cold) and collected after ABSP treatment. Then cell pellets were suspended in the Radioimmunoprecipitation assay (RIPA) lysis (Bioworld technology, Minnesota, US) for 30 min. After centrifugation (12,000 rpm) for 5 min, the supernatant was collected. A BCA protein quantitative kit (Biotopped, Beijing, China) was used to evaluate the total protein content in the sample. According to quantitative results, different groups were mixed with 5 times protein loading buffer by equal quality. After centrifugation, the samples were electrophoresed on SDS polyacrylamide gels and transferred to polyvinylidene fluoride membranes. After being incubated with the primary antibodies for 1 h (at room temperature), the membranes were rinsed with PBS (containing 0.2% Tween 20) and incubated with the corresponding secondary antibodies for another 1 h. With the chemiluminescent method, the proteins were finally detected after washing. The signals were detected and imaged using an ECL-Plus detection system (GE Healthcare, South Plainfield, United States).

### Statistical Analysis

A one-way fixed-effects analysis of variance (ANOVA) test was performed using statistical software (SPSS version 18.0, SPSS Inc., Chicago, IL, United States). All trials were done in triplicate, and the statistical means and standard deviations were calculated and shown.

## Results and Discussions

### Protection of Sea Buckthorn Proanthocyanidins on the Mitochondrial Membrane Potential in H_2_O_2_-Induced Damage RAW 264.7 Cells

**FIGURE 1:**
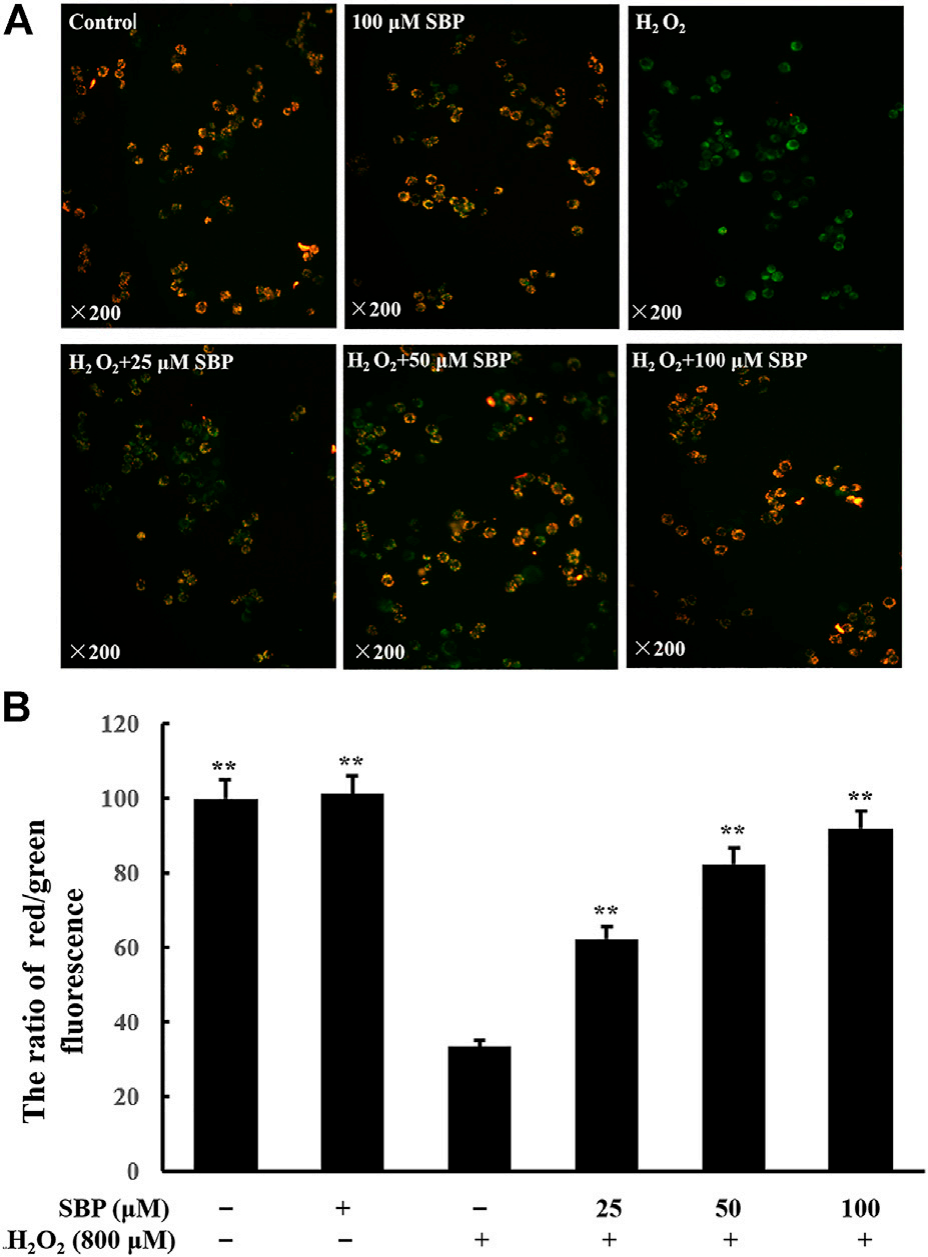
Effects of SBP on mitochondrial membrane potential in H_2_O_2_-induced RAW 264.7 cells. RAW 264.7 cells were pretreated with SBP for 4 h, then treated with H_2_O_2_ (800 µM) for 12 h, and then dyed with 5 µM JC-1 for 1.5 h at 37°C in the dark. **(A)**: Qualitative analysis with an inverted fluorescence microscope (×200); **(B)**: Quantitative analysis with a multimode microplate reader at 485 nm excitation, 538 nm (green) and 585 nm (red) emissions for JC-1 staining. *p < 0.05, and **p < 0.01 versus H_2_O_2_-treated groups.

Mitochondria are responsible for redox homeostasis. They are cell organelles that are susceptible to oxidative damage. H_2_O_2_ is a known suppressing factor in mitochondrial dysfunction. The decrease in mitochondrial membrane potential indicates mitochondrial dysfunction and apoptosis (Kim, 2005). JC-1 can selectively enter mitochondria to form a monomer, which indicates the mitochondrial potential (MMP) in cells. If MMP collapses during apoptosis, the fluorescence of JC-1 changes from red to green. Thus, the green and red ratio of JC-1 indicates the variation of MMP (Taylor et al., 2003). As shown in Figure 1A, compared with the control group, the fluorescence of JC-1 of the H_2_O_2_ group turned green, which indicated that the MMP was significantly decreased. Meanwhile, the SBP groups showed less green emissions and more red emissions, which indicated that there is less reduction in the MMP.

To analyze this quantitatively, the ratio of red/green fluorescence was analyzed. Referring to Figure 1B, compared with the control group, there was a significant decrease in the ratio in the H_2_O_2_ group, which indicated a reduction in mitochondrial function. This ratio increased in the SBP groups in a concentration-dependent manner. These results indicated that SBP could protect the cells against H_2_O_2_-induced mitochondrial dysfunction.

### Sea Buckthorn Proanthocyanidins Protects Mitochondria Microstructure From H_2_O_2_-Induced Damage in RAW 264.7 Cells

**FIGURE 2:**
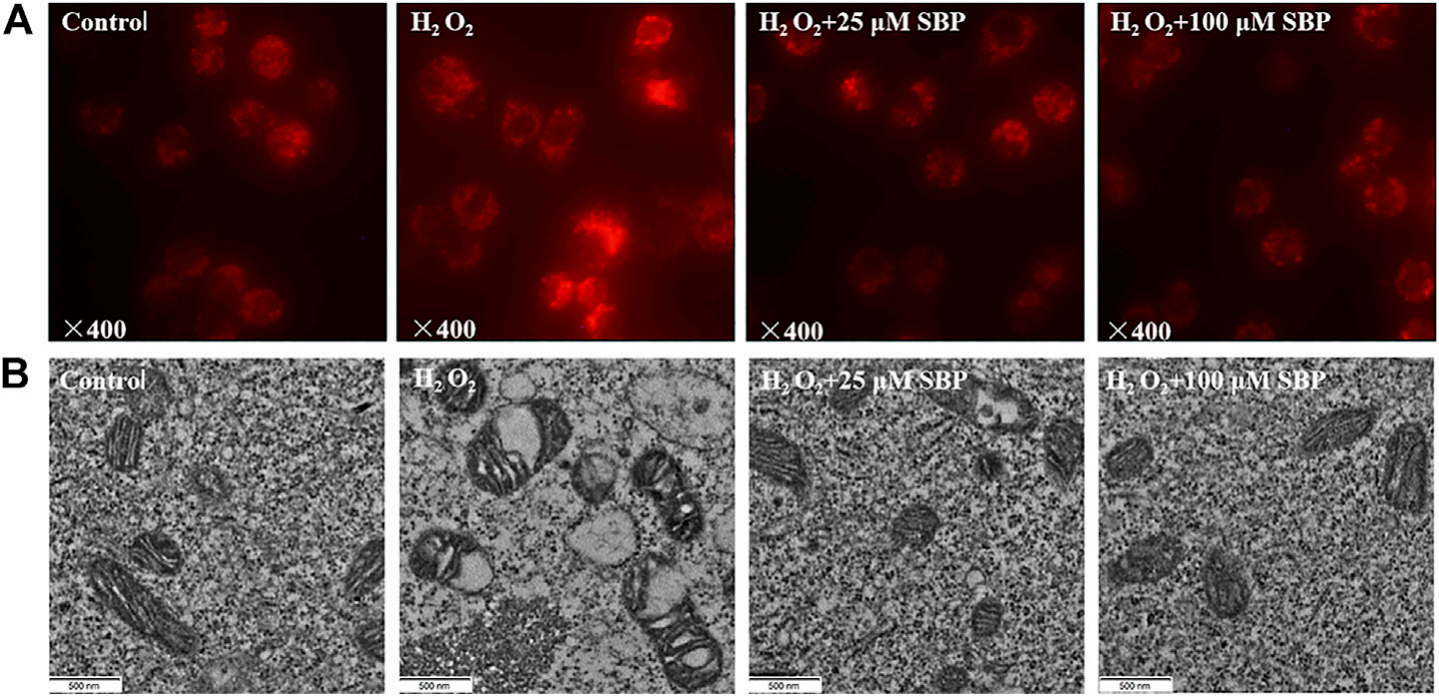
Effects of SBP on morphology of mitochondrial of RAW 264.7 cells. **(A)**: Representative images show morphology of mitochondria. Mitochondrial morphology was visualized after staining the cells with MitoTracker Red and images were captured under fluorescence microscope; **(B)**: Transmission-electron-microscopy (TEM) images of mitochondria in RAW 264.7 cells (×21,000).

Since mitochondrial membrane potential is critical for maintaining mitochondrial bioenergetics and dynamics, we investigated the effects of SBP in mitochondrial morphology of RAW264.7 cells under disturbed redox homeostasis. Mitochondria are the major endogenous source of oxidative stress (Daverey and Agrawal, 2016). Hence, fluorescent probe Mito-Tracker Red dye was used to analyze the changes in mitochondria morphology induced by oxidative stress in RAW 264.7 cells. As shown in Figure 2A, the distribution of mitochondria changed under fluorescent microscopy. The mitochondria of normal cells were evenly distributed and had a clear tubular shape. Compared with the control group, the mitochondria morphology of the H_2_O_2_ group became swollen, fuzzy, uneven, and aggregated. A transmission electron microscope (TEM) was used to observe the morphological differences of mitochondria among different groups. Referring to Figure 2B, it showed that mitochondria transformed from the typical tubular shape into swollen and fragmented structures under oxidative stress. Furthermore, mitochondrial autophagy was also observed. Remarkably, the SBP treatment alleviated the H_2_O_2_-induced mitochondrial swelling in RAW 264.7 cells, indicating that SBP had protective effects on mitochondria. These results suggest that SBP prevents mitochondria damage induced by oxidative stress in RAW 264.7 cells.

### Sea Buckthorn Proanthocyanidins Inhibited Mitochondrial Swelling via Permeability Transition Pore Induction in H_2_O_2_-Induced Damage RAW 264.7 Cells

**FIGURE 3:**
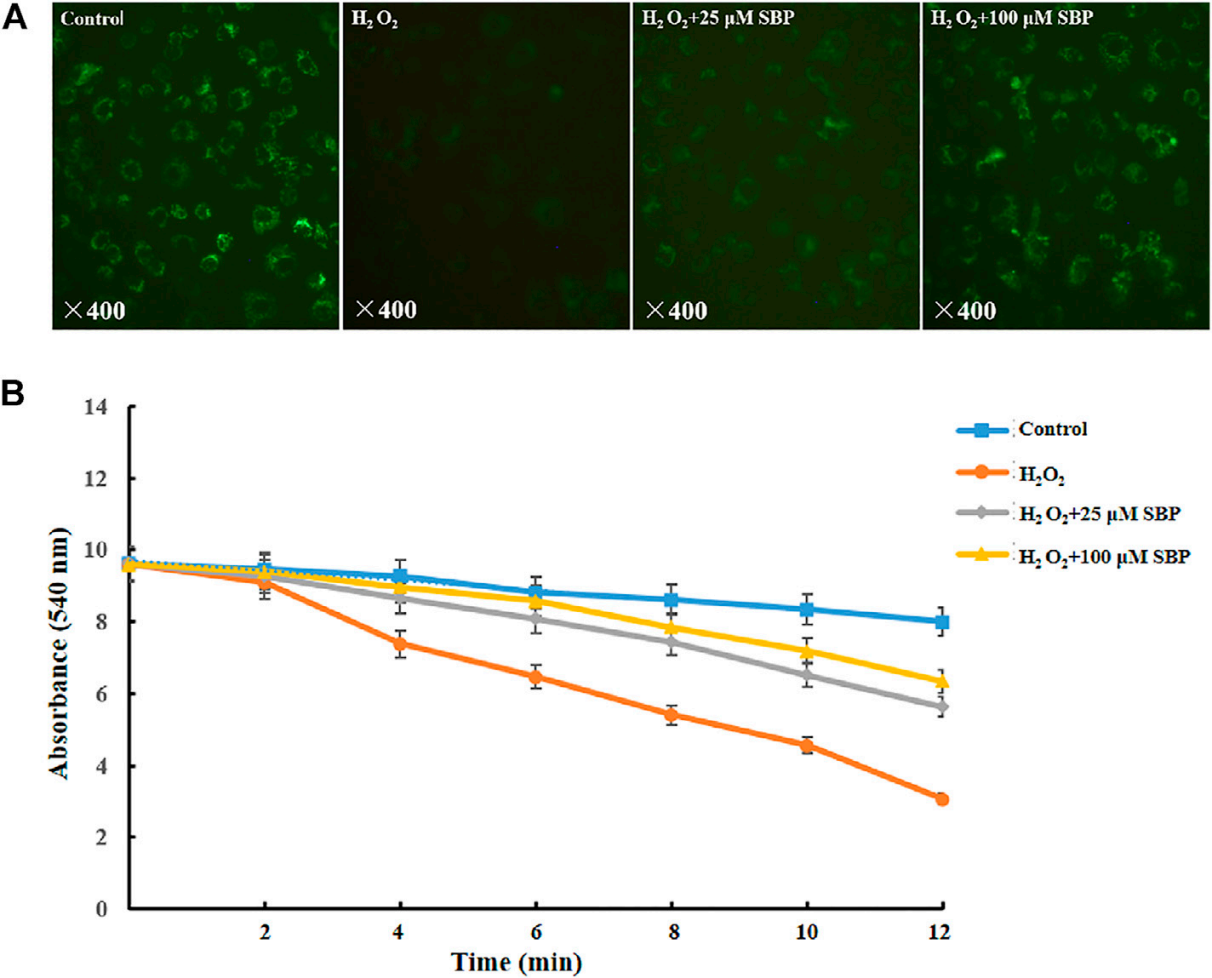
Effects of SBP on morphology of mitochondrial permeability and swelling. **(A)** Mitochondrial permeability transition pore of RAW 264.7 cells. The green and red components of the images indicate calcein fluorescence. **(B)** Mitochondrial swelling of RAW 264.7 cells.

Firstly, we measured the level of the mPTP opening at a cellular level by mPTP Assay Kit (C2009S, Beyotime Biotechnology, Shanghai, China). The degree of mPTP opening was judged according to the intensity of Calcein green fluorescence in mitochondria. If the green fluorescence was stronger, the opening of mPTP was lower; conversely, the weaker green fluorescence was, the opening of mPTP was higher. Referring to Figure 3A, compared with the control groups, the H_2_O_2_ group showed a significant reduction in green fluorescence intensity, indicating that the opening of mPTP was obviously enhanced. However, the SBP group significantly restored the green fluorescence intensity, indicating that SBP effectively inhibited the opening of mPTP. Under many pathological conditions, as the mitochondrial permeability transition pore (mPTP) opens, it leads to mitochondrial swelling, reduced oxidative phosphorylation capacity, and cell necrosis or apoptosis (Bernardi, 1999).

After that, we measured the mPTP opening at the mitochondrial level and mitochondrial swelling through *in vitro* separation. In the mitochondria-CA^2+^ system, referring to Figure 3B, compared with the control group, the H_2_O_2_ group showed a significant decrease in absorbance at 540 nm. This indicated that H_2_O_2_ led to mitochondrial swelling and damage of mitochondrial function. Besides, the SBP groups showed less reduction in the absorbance at 540 nm than the H_2_O_2_ group in a concentration-dependent manner. This suggested that SBP improved mitochondrial damage and dysfunction caused by oxidative stress. These results further supported the TEM observation.

### Sea Buckthorn Proanthocyanidins Promoted the Biosynthesis of the Mitochondria in H_2_O_2_-Induced Damage RAW 264.7 Cells

**FIGURE 4:**
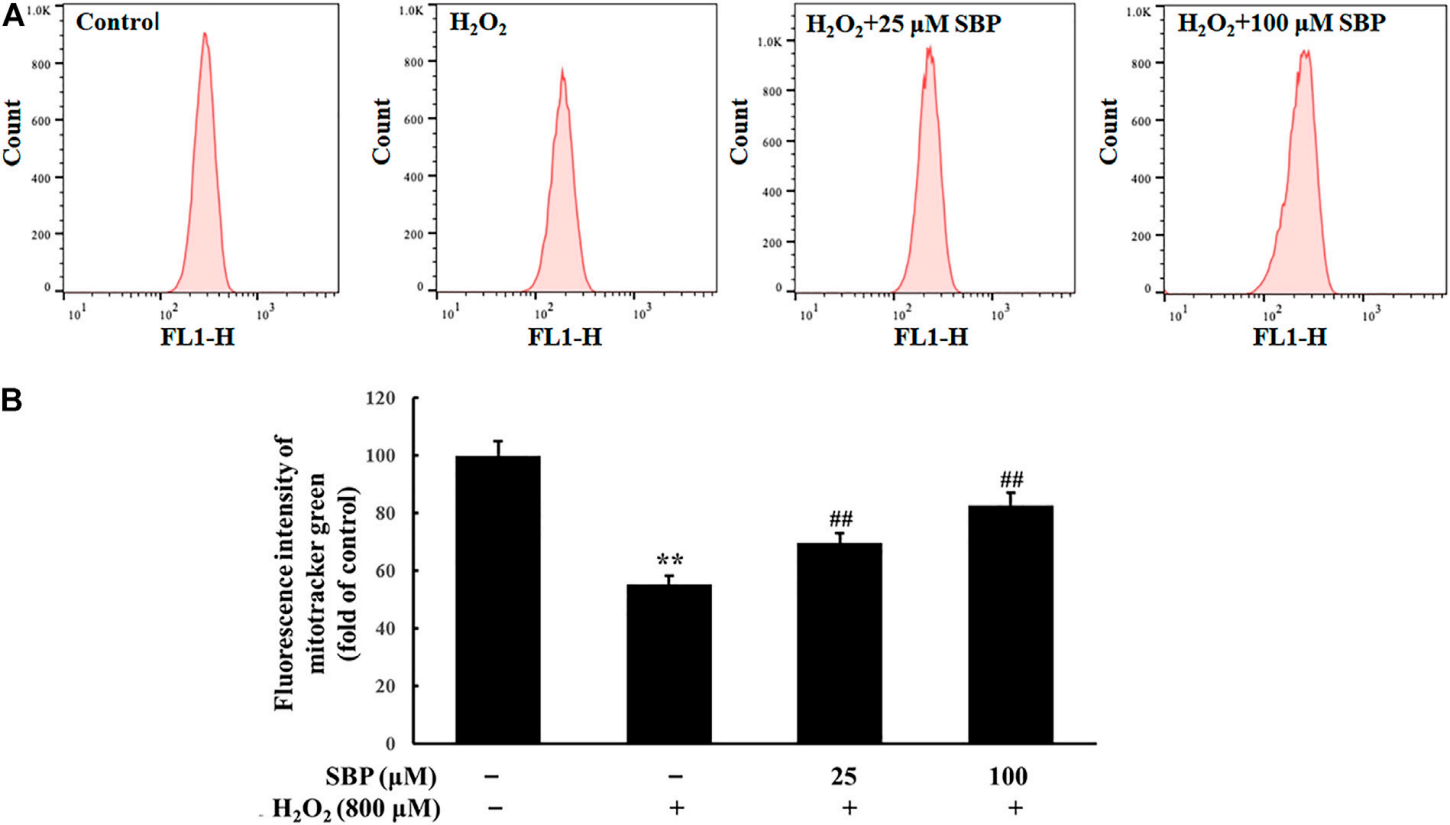
Effect of SBP on the mitochondrial mass in RAW 264.7 cells. **(A)** Representative flow cytometric histograms of each group. **(B)** The relative fluorescence intensities of RAW 264.7 cells treated with Mito-Tracker green. **p < 0.01 compared to the control group; ##*p* < 0.01 compared to the H_2_O_2_-treated group.

Mitochondrial mass was measured by fluorescent probe Mito-Tracker Green through flow cytometry. As shown in Figure 4, compared with the control group, the H_2_O_2_ group showed a significant decrease in the mean fluorescence intensity. Conversely, the SBP groups showed less decrease in the mitochondrial mass than the H_2_O_2_ group in a concentration-dependent manner. Under oxidative stress, the mitochondria synthesis of cells was inhibited, which affected their energy metabolism and antioxidant capacity. This result showed that SBP improved mitochondrial synthesis, significantly protecting the mitochondrial density. Therefore, they suggested that SBP may affect mitochondrial energy production and enzyme activities related to energy metabolism.

### Sea Buckthorn Proanthocyanidins Improved the Energy Metabolism of the Mitochondria in H_2_O_2_-Induced Damage RAW 264.7 Cells

**FIGURE 5:**
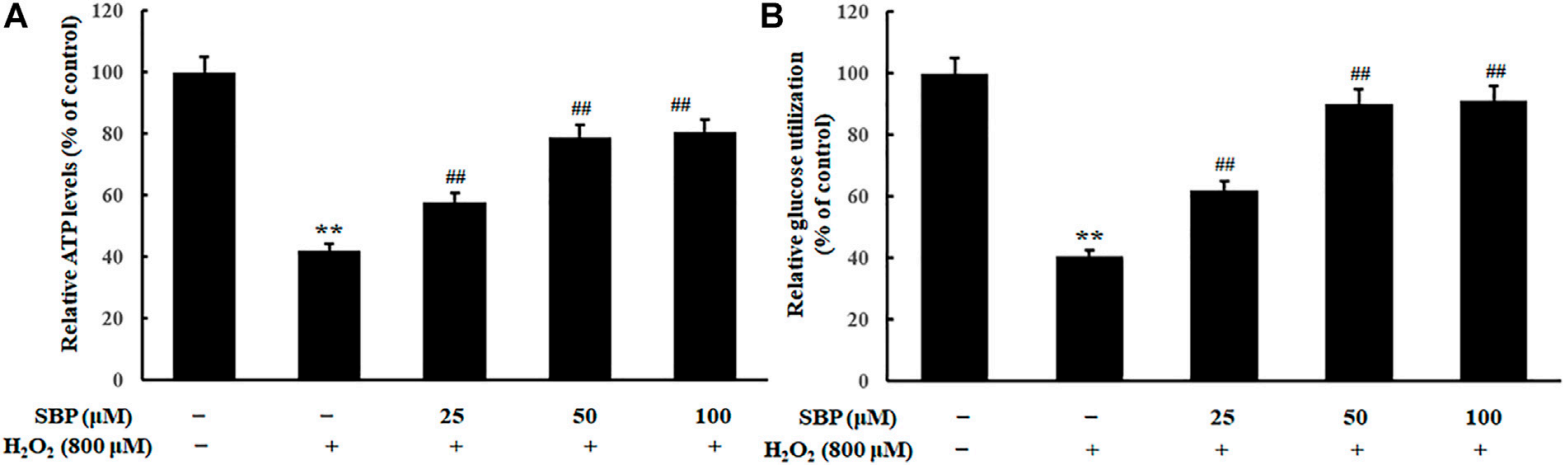
Effect of SBP on the mitochondrial energy metabolism in RAW 264.7 cells. **(A)** Relative quantifying intracellular ATP levels in mitochondria after treatment with SBP. Representative flow cytometric histograms of each group. **(B)** Relative quantifying cellular glucose utilization in mitochondria after treatment with SBP. ATP content and glucose utilization were measured after 24 h treatment. **p < 0.01 compared to the control group; ##*p* < 0.01 compared to the H_2_O_2_-treated group.

As shown in Figure 5, compared with the control group, the H_2_O_2_ group showed a significant reduction in relative ATP levels. In contrast, the SBP groups showed a positive correlation between the relative ATP levels and the SBP concentration. ATP is produced in mitochondria to provide energy. Mitochondria are important organelles, which are responsible for cellular respiration. This is because they are responsible for synthesizing ATP required for daily life (Hong et al., 2020). Relative ATP levels were analyzed to determine whether SBP was effective in preventing the reduction of the mitochondrial enery metabolism in H_2_O_2_ treated RAW 264.7 cells.

Apart from that, relative glucose utilization is an important indicator of oxidative damage. Hence, we compared the relative glucose utilization of H_2_O_2_ treated Raw 264.7 cells with and without the presence of SBP. Compared with the control group, the H_2_O_2_ group showed a significant reduction in relative glucose utilization. Moreover, the SBP groups showed less reduction in the relative glucose utilization when compared with the H_2_O_2_ group in a concentration-dependent manner. This result suggested that SBP helped protect Raw 264.7 cells from oxidative damages.

### Sea Buckthorn Proanthocyanidins Regulate Nrf2-Regulated Signal Pathway in H_2_O_2_-Induced Damage RAW 264.7 Cells

**FIGURE 6:**
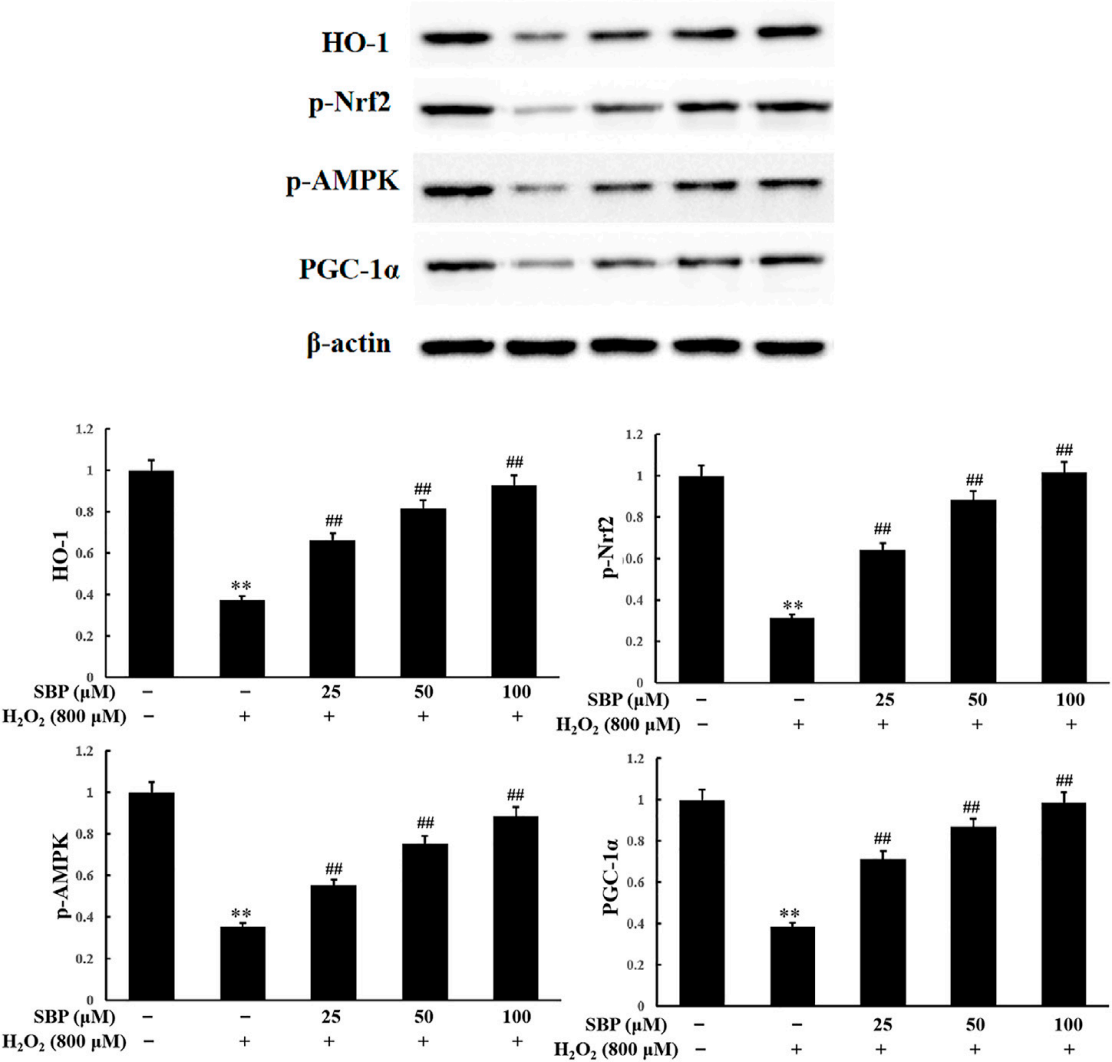
Effect of SBP on the protein expression levels of HO-1, phosphorylated Nrf2, AMPK and PGC-1α. After that, cell lysates were collected and subjected to western blotting analyses. Data are calculated from three independently replicated experiments. *p* < 0.01 compared to the control group; *p* < 0.01 compared to the H_2_O_2_-treated group.

To investigate the inhibitory effect of SBP on H_2_O_2_ oxidative stress and its relationship with the activation of the Nrf2/HO-1 signaling pathway, the level of Nrf2 and HO-1 expression was analyzed. As shown in Figure 6, the result of the Western Blot analysis showed that SBP significantly suppressed the reduction in HO-1 expression induced by H_2_O_2_ in RAW 264.7 cells. This suggested that SBP increased the activity of HO-1 under oxidative stress conditions. Since Nrf2 is needed to be phosphorylated and then transported to the nucleus to initiate the expression of antioxidant proteins, such as HO-1, we further investigated whether SBP could regulate the nuclear transport of p-Nrf2. Compared with the control group, SBP reduced the suppression of p-Nrf2 induced by H_2_O_2_ in a concentration-dependent manner. These results suggested that SBP mediated the induction of HO-1 through nuclear translocation of Nrf2 in RAW 264.7 cells.

HO-1 has various physiological functions, such as antioxidation, anti-apoptosis, anti-inflammation, and immune regulation. Nrf2 is the key transcription factor for intracellular resistance to oxidative stress, which can be activated by antioxidants. Nrf2 and PGC-1α cotranscript mitochondrial proteins and mtTFA, controlling mitochondrial DNA replication and transcription. This influences the generation of mitochondria (Staniek and Nohl, 2000). The activity of AMPK is closely related to the phosphorylation level of Nrf2. Both affect mitochondrial function. Pomegranate extract activated the AMPK/Nrf2 signaling pathway and alleviated oxidative stress and mitochondrial dysfunction in hypertensive rats (Sun et al., 2016). Moreover, butin also activated the AMPK/Nrf2 signaling pathway, reducing oxidative stress, improving mitochondrial function, and increasing HO-1 expression (Duan et al., 2017). As shown in Figure 6, compared to the control group, the H_2_O_2_ group showed a significant reduction in p-AMPK expression. The SBP groups showed an increase in p-AMPK expressions when compared with the H_2_O_2_ group. There was a positive correlation between the SBP concentration and p-AMPK expressions. These results indicated that SBP significantly improved the phosphorylation of Nrf2 by activating p-AMPK expression.

PGC-1α plays an important role in the mitochondrial synthesis and redox homeostasis. SBP were shown to protect the number of mitochondria and AMPK. AMPK is an upstream signaling molecule of PGC-1α. Hence, we analyzed the expression level of PGC-1α. Referring to Figure 6, compared with the control group, the H_2_O_2_ group showed a significant reduction in PGC-1α expression. The SBP groups showed increase in PGC-1α expression in a concentration-dependent manner. These results suggested that SBP can activate PGC-1α expression, by promoting the generation of mitochondria in cells. This alleviated H_2_O_2_-induced cellular oxidative stress and damage. These results were consistent with the previous TEM and fluorescence results.

## Conclusion

As to conclude, this study demonstrated that SBP played an important role in the protection of RAW264.7 cells under oxidative stress. The mechanism included the regulation of mitochondrial energy metabolism, generation, and morphological and functional properties. SBP increased mitochondrial membrane potential, inhibited mPTP opening, relieved mitochondrial swelling, and increased mitochondrial generation metabolism. Thereby, it improved oxidative damage and protected mitochondrial function. SBP were shown to activate the AMPK-PGC-1α-Nrf2 pathway. This study provided a deeper understanding of the mechanism behind the protective effect of SBP on mitochondria. These results will lay the foundation for the application of sea buckthorn proanthocyanidins as an antioxidant functional food.

## Data Availability

The original contributions presented in the study are included in the article/Supplementary Materials, further inquiries can be directed to the corresponding author.

## Abbreviations

SBP, sea buckthorn proanthocyanidins; MMP, mitochondrial membrane potential; mPTP: mitochondrial permeability transition pore.
