# Supplementary material for: Sea Buckthorn Proanthocyanidins are the Protective Agent of Mitochondrial Function in Macrophages Under Oxidative Stress
Source: Front Pharmacol. 2022 Jul 8;13:914146. doi: 10.3389/fphar.2022.914146 (PMC9307083; doi:10.3389/fphar.2022.914146)
Supplement: Supplementary file 1 [file DataSheet1.DOC]

Full scan of the entire original gels

(Figure 6 : We used the first 5 columns of strips from the left:)


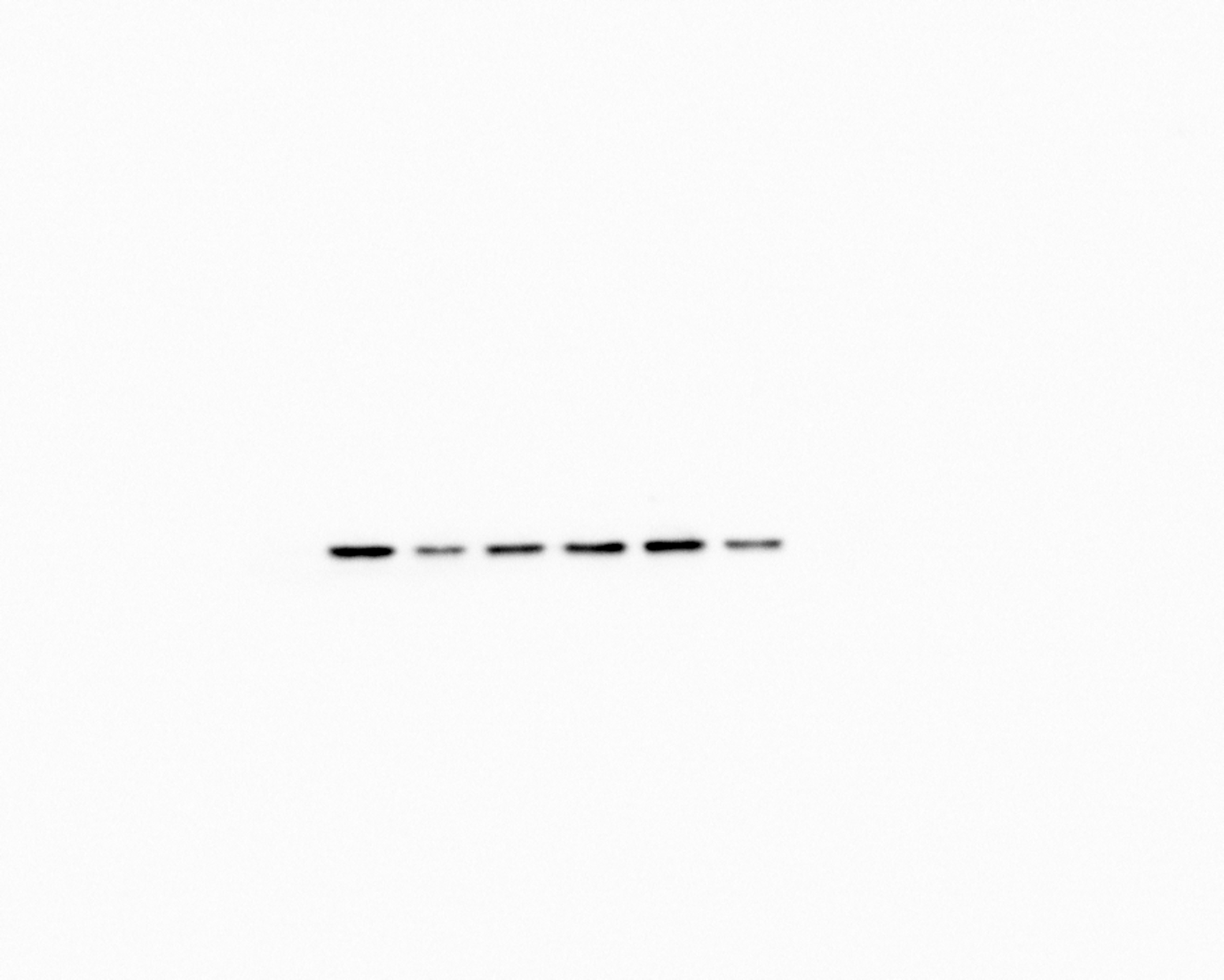


HO-1


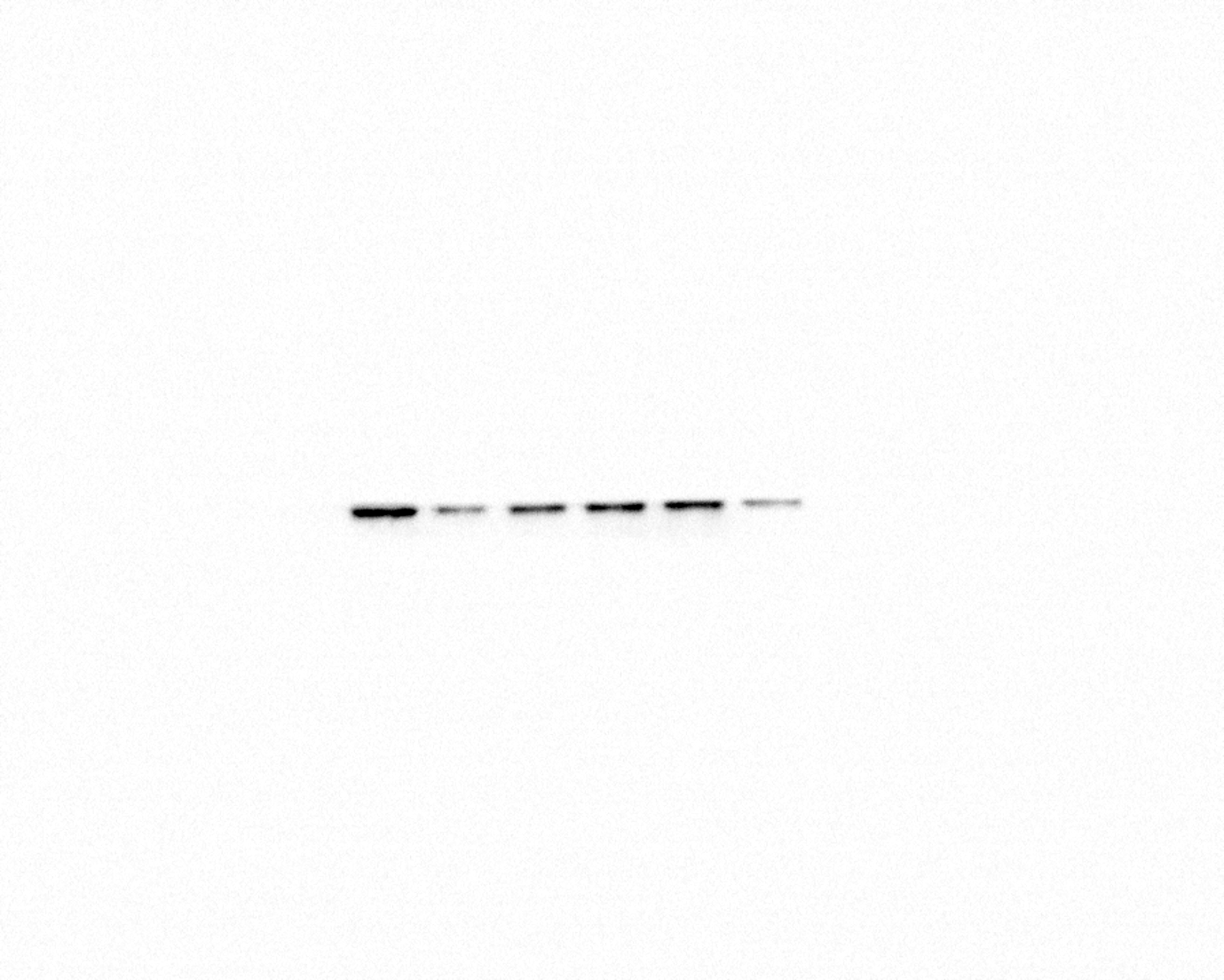


1. AMPK


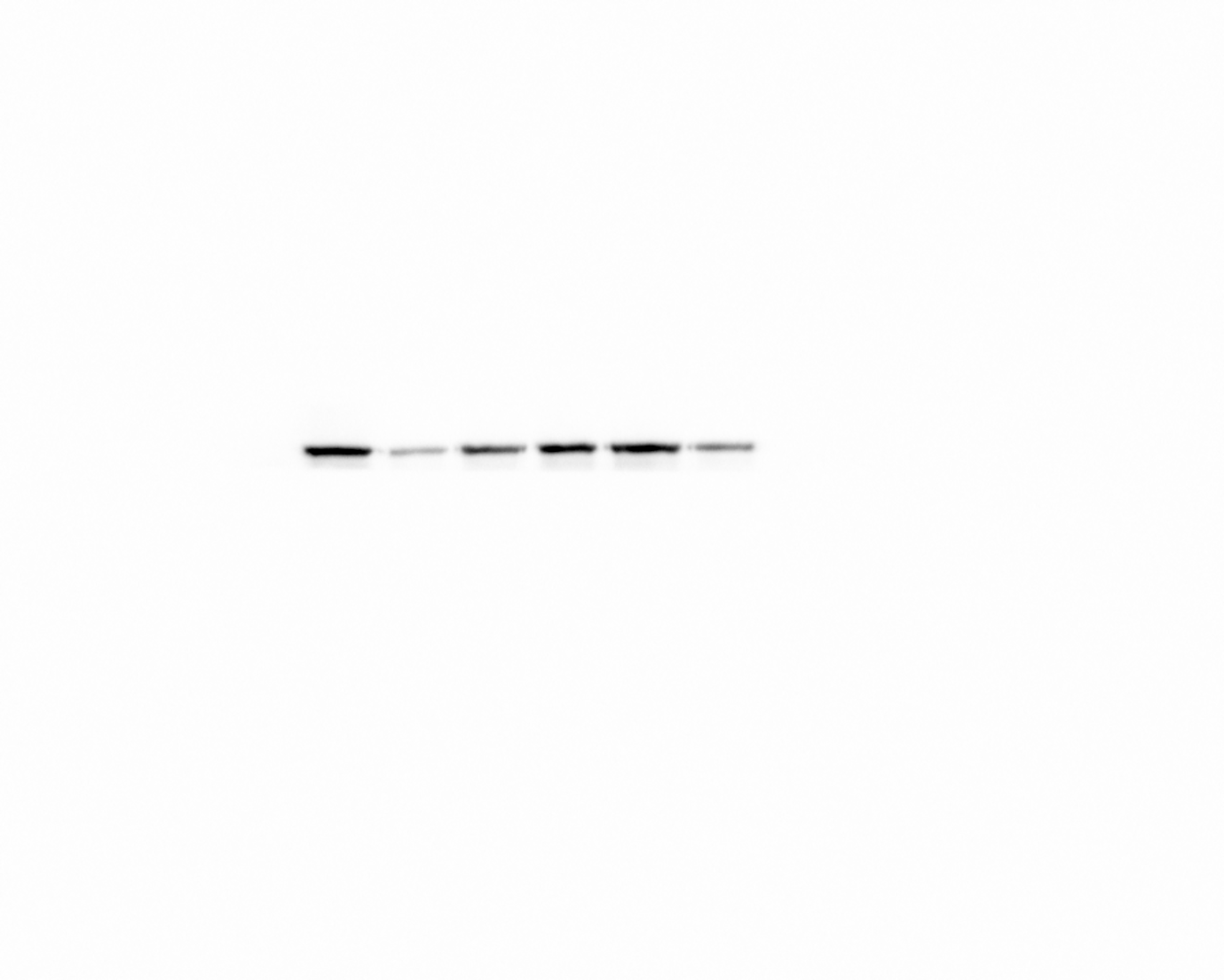


P-NRF2


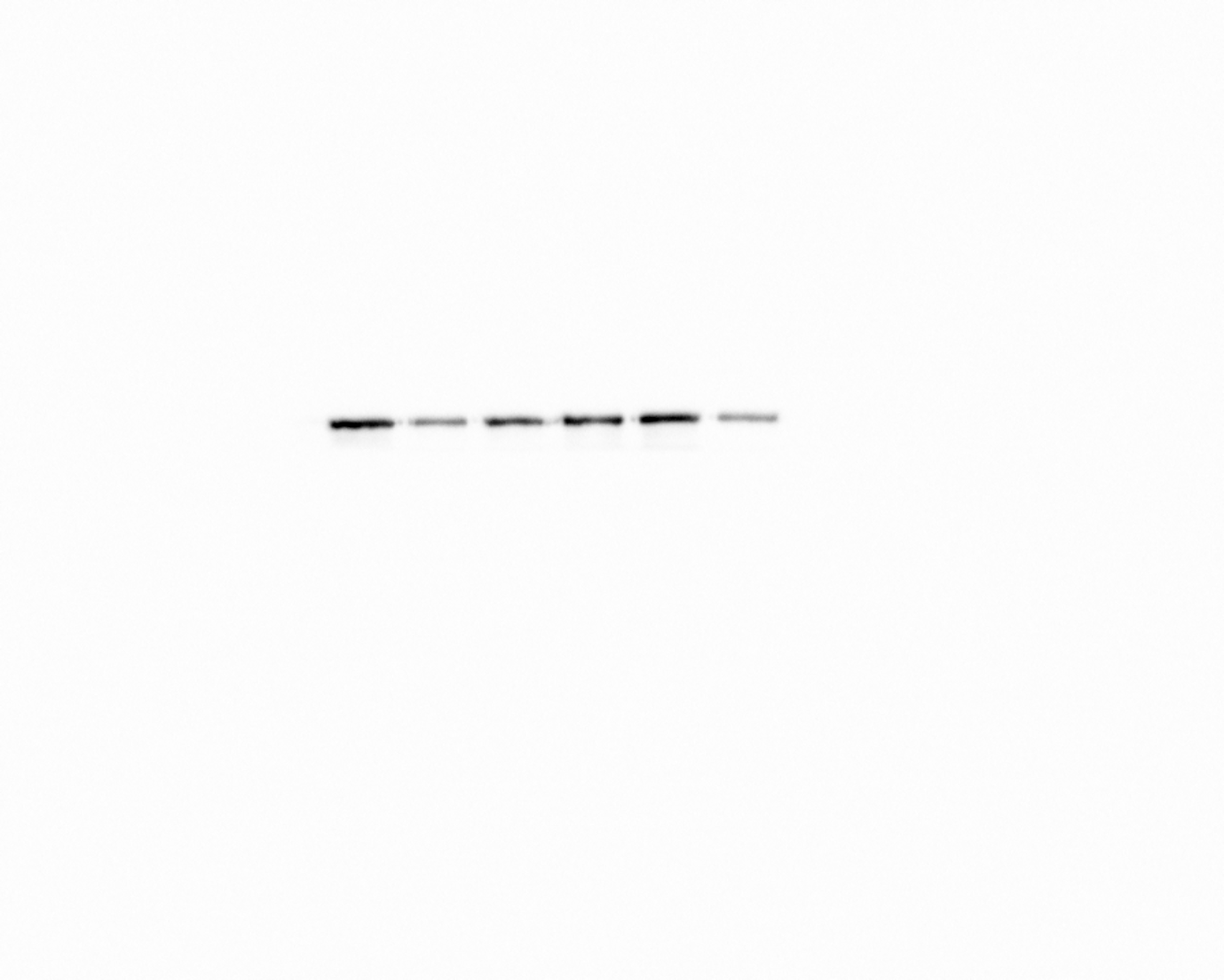


PGC-1α


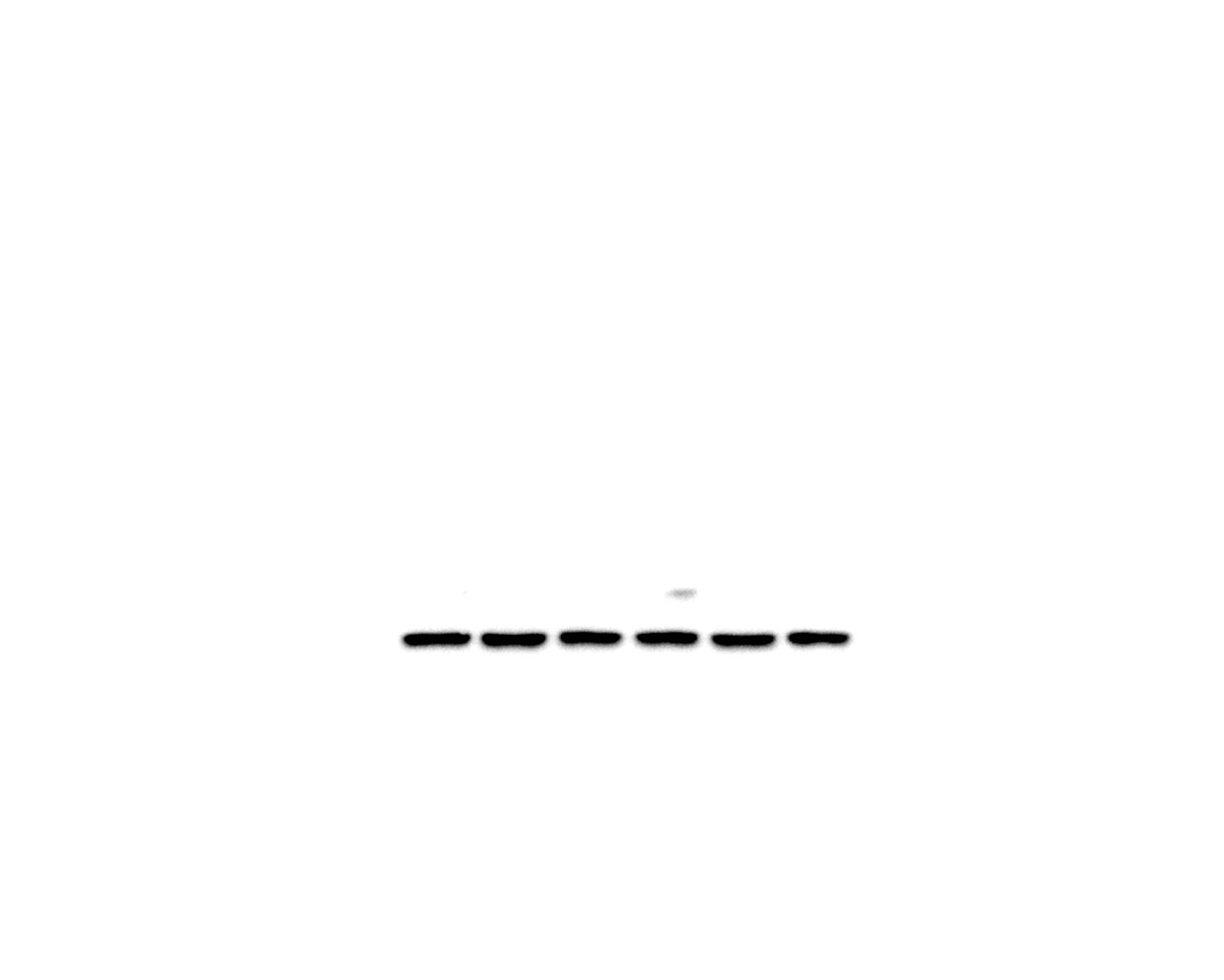


β-actin
